# Supplementary material for: Rho-dependent transcription termination proceeds via three routes
Source: Nat Commun. 2022 Mar 29;13:1663. doi: 10.1038/s41467-022-29321-5 (PMC8964686; doi:10.1038/s41467-022-29321-5)
Supplement: Supplementary file 1 — Supplementary Information [file 41467_2022_29321_MOESM1_ESM.pdf]

Supplementary Information

Rho-dependent transcription termination proceeds via three routes

Eunho Song[1], Heesoo Uhm[1,4], Palinda Ruvan Munasingha[2], Seungha Hwang[3], Yeon-Soo Seo[2], Jin Young Kang[3]*, Changwon Kang[2]*, Sungchul Hohng[1]*

[1] Department of Physics and Astronomy, and Institute of Applied Physics, Seoul National University, Seoul 08826, Republic of Korea
[2] Department of Biological Sciences, Korea Advanced Institute of Science and Technology, Daejeon 34141, Republic of Korea
[3] Department of Chemistry, Korea Advanced Institute of Science and Technology, Daejeon 34141, Republic of Korea
[4] Present address: Department of Physics, University of Oxford, Oxford OX1 3PU, UK
* Corresponding authors: shohng@snu.ac.kr; ckang@kaist.ac.kr; jykang59@kaist.ac.kr

Contents

Supplementary Figure 1: Single-molecule imaging
Supplementary Figure 2: Timings of fluorophore photobleaching and RNA release
Supplementary Figure 3: Detailed explanation of fluorescence time traces
Supplementary Figure 4: Portions of recycling and decomposing terminations in holistic assays
Supplementary Figure 5: Representative fluorescence time traces of stand-by and catch-up assays
Supplementary Figure 6: Stability of ρ·RNAP complex
Supplementary Figure 7: Masking efficiencies of ρ mutants
Supplementary Figure 8: *In vitro* bulk transcription assays
Supplementary Figure 9: Background termination of five ρ-dependent terminators
Supplementary Figure 10: Purification of ρ mutants
Supplementary Table 1: Oligonucleotide sequences
Supplementary Table 2: Termination efficiencies
Supplementary Table 3: Readthrough and termination timings (Fig. 3d-e)
Supplementary Table 4: Statistics for readthrough and termination timings (Fig. 3d)

Abbreviations: BSA, bovine serum albumin; EC, elongation complex; nt, nucleotides; NTP, ribonucleotide(s); PIFE, protein-induced fluorescence enhancement; TE, termination efficiency

Supplementary Figures

Supplementary Figure 1 | Single-molecule imaging. a-b. Cy3-labeled RNA images (black spots) were taken just before (left) and 600 s after (right) injection of NTP+ρ (**a**) or ρ alone (**b**). The scale bar is 5 μm long. On the right images in **a** and **b**, blue circles indicate the spots that lost Cy3-RNA after the injection, and red circles indicate those that did not. The red circles on the right image in **a** could be transcriptionally inactive complexes or the active complexes where termination or run-off has not occurred yet.

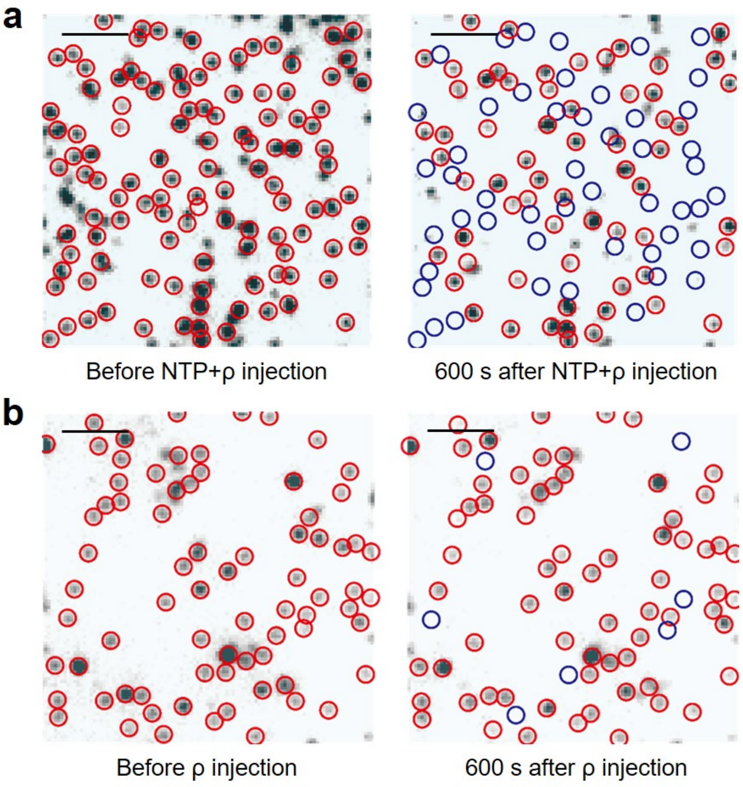

Supplementary Figure 2 | Timings of fluorophore photobleaching and RNA release. We measured photobleaching time with injection of ρ alone without NTPs (black, n = 110 molecules), and RNA release time with injection of both ρ and NTPs (red, n = 340 molecules). The fractions of molecules with Cy3 fluorescence in y-axis are plotted as a function of time after the injection in x-axis, and fit to single exponential functions. Source data are provided in a Source Data file.

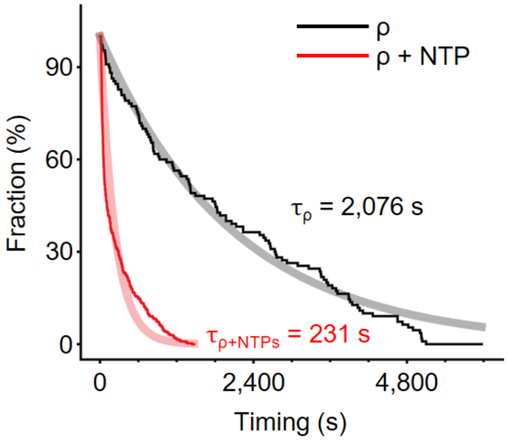

Supplementary Figure 3 | Detailed explanation of fluorescence time traces. a-c. Detailed fluorescence time traces for readthrough (**a**), recycling termination (**b**), and decomposing termination (**c**). Vertical line in each time trace indicates timing of NTP addition. Below the traces, gray time ribbon shows the absence (blank) and presence (filled) of NTPs. Green time ribbon shows Cy3 fluorescence changes. Red time ribbon shows Cy5 fluorescence changes. At the bottom, schematic diagrams of the molecular events drawn from the fluorescence changes are shown.

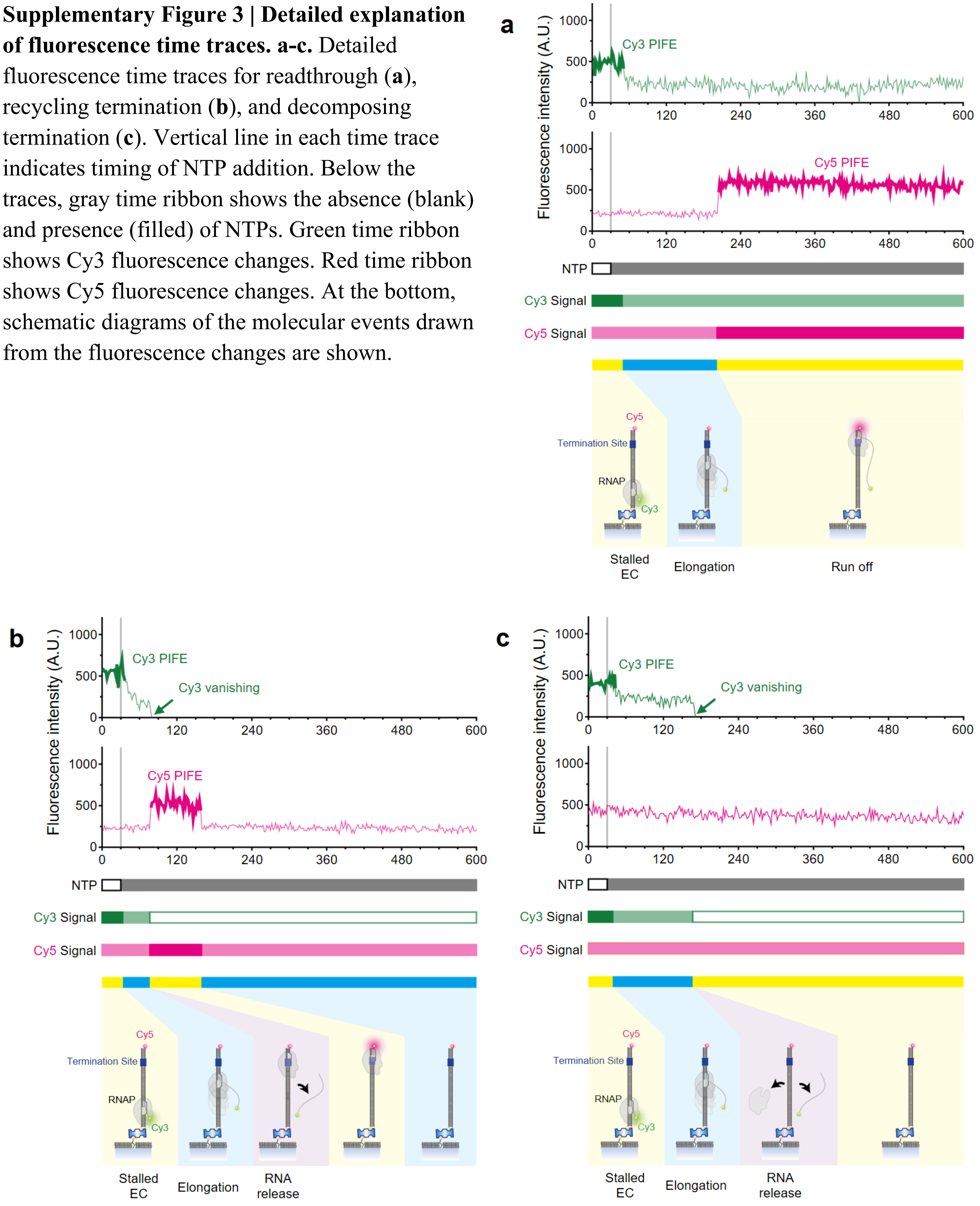

Supplementary Figure 4 | Portions of recycling and decomposing terminations in holistic assays. Portions of recycling TEs (solid) and decomposing TEs (open) measured in holistic assays in y-axis are plotted against ρ concentrations (5 to 200 nM) in x-axis. An error bar represents standard deviation of mean from $n \geq 3$ independent experiments each with >50 molecules. Source data are provided in a Source Data file.

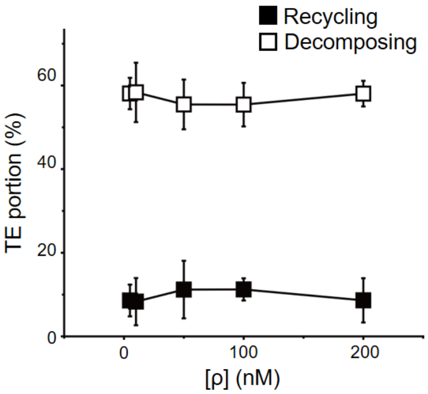

Supplementary Figure 5 | Representative fluorescence time traces of stand-by and catch-up assays. a-b. Representative fluorescence time traces of Cy3 (green) and Cy5 (red) at Cy3 excitation (top) and Cy5 excitation (bottom) in stand-by assay (**a**) and catch-up assay (**b**). NTP+ρ were injected at 30 s (gray lines) after the fluorescence monitoring starts.

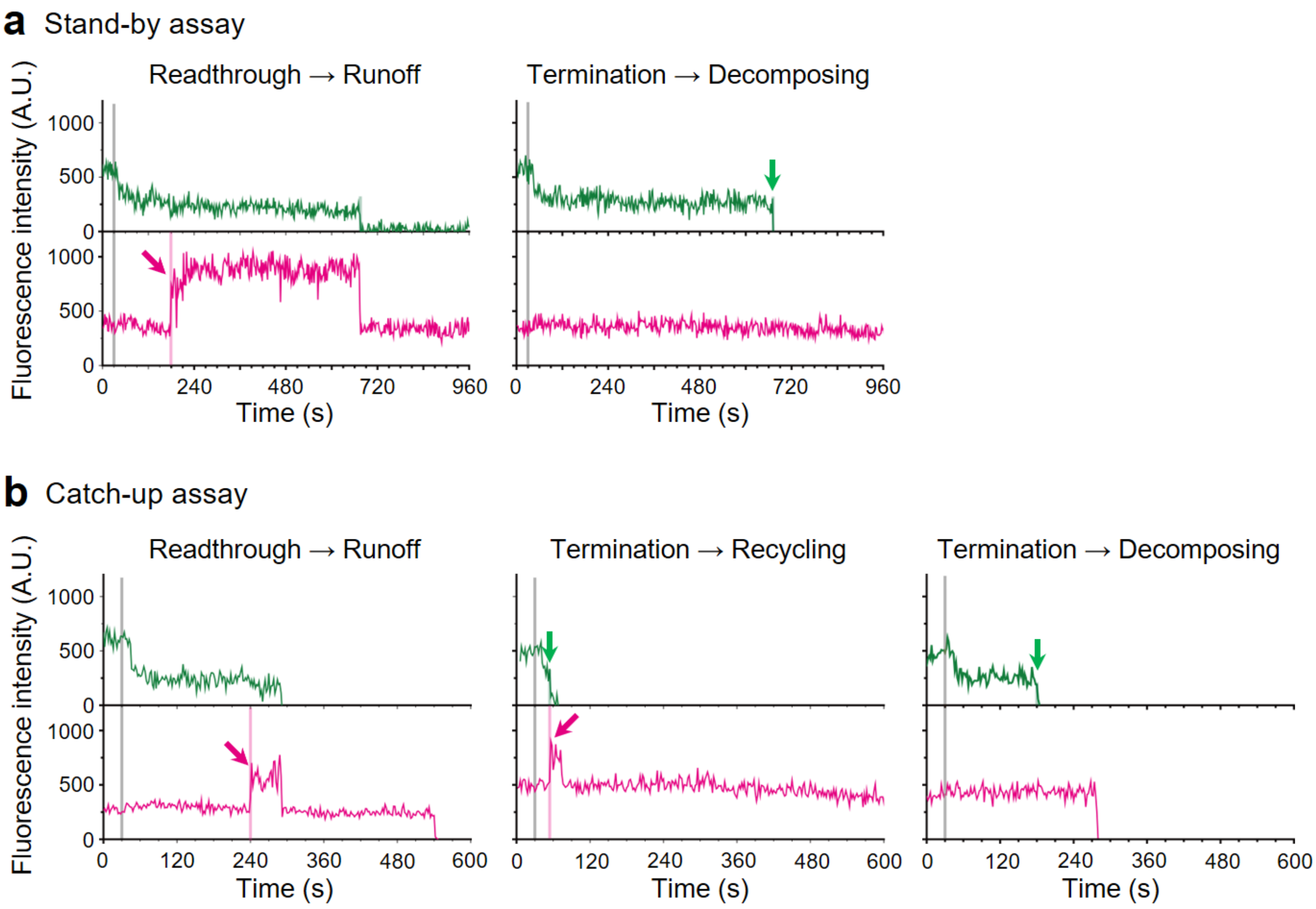

Supplementary Figure 6 | Stability of ρ·RNAP complex. Normalized TEs, i.e. ρ-dependent TEs divided by (100% - background TE), of *mgtA* terminator by pre-bound ρ in y-axis are plotted against the interval time between ρ wash-out and NTP injection without (open, 693 s) and with (solid, 1,803 s) NusA and NusG in x-axis, and fit to single exponential functions. Source data are provided in a Source Data file.

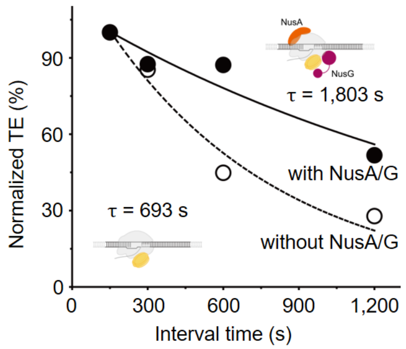

Supplementary Figure 7 | Masking efficiencies of ρ mutants. a. Scheme for the stand-by single-molecule assay modified for estimating the masking efficiencies of ρ mutants. **b-c.** The ρ-dependent decomposing TEs were measured with sequential incubation of a ρ mutant and wild-type (masking) or with wild-type ρ alone (stand-by) in the modified stand-by assays without (**b**) or with (**c**) NusA and NusG. An error bar represents standard deviation of mean from $n \geq 3$ independent experiments each with >50 molecules. Source data are provided in a Source Data file.

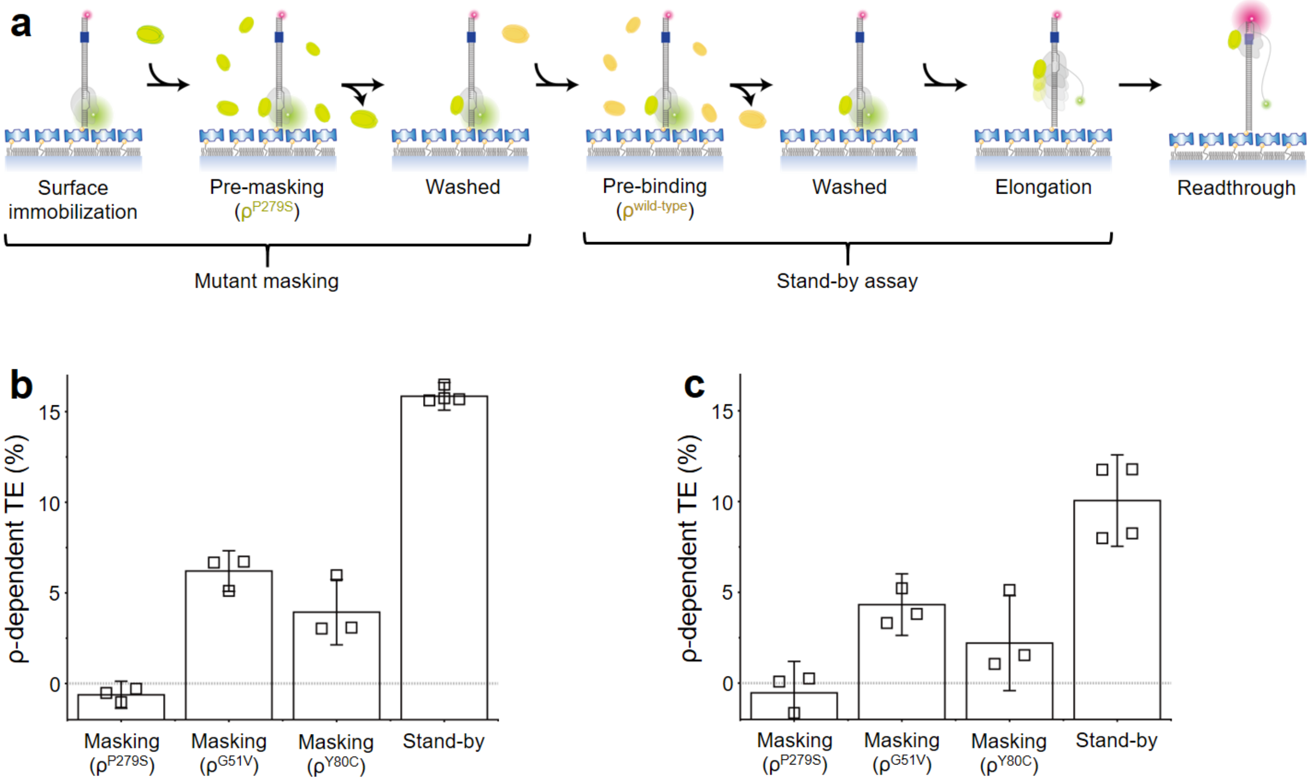

Supplementary Figure 8 | *In vitro* bulk transcription assays. a-e. Bulk transcription reactions were carried out *in vitro* with a template harboring *mgtA* terminator (**a**), *λtR1* terminator (**b**), *trp-t'* terminator (**c**), *rho* terminator (**d**), or *ribB* terminator (**e**) at 0, 15, 30, or 60 nM ρ and in the absence or presence of NusA and NusG (NusA/G) for 10 min. The run-off (RO) and terminated transcript bands (vertical lines) are marked to the right of each gel image. RNA size markers from 100 to 314 nucleotides (nt) were used in 6%-polyacrylamide urea gel electrophoresis. The raw and ρ-dependent (ρ-dep) TEs are shown below each gel image. Source data are provided in a Source Data file.

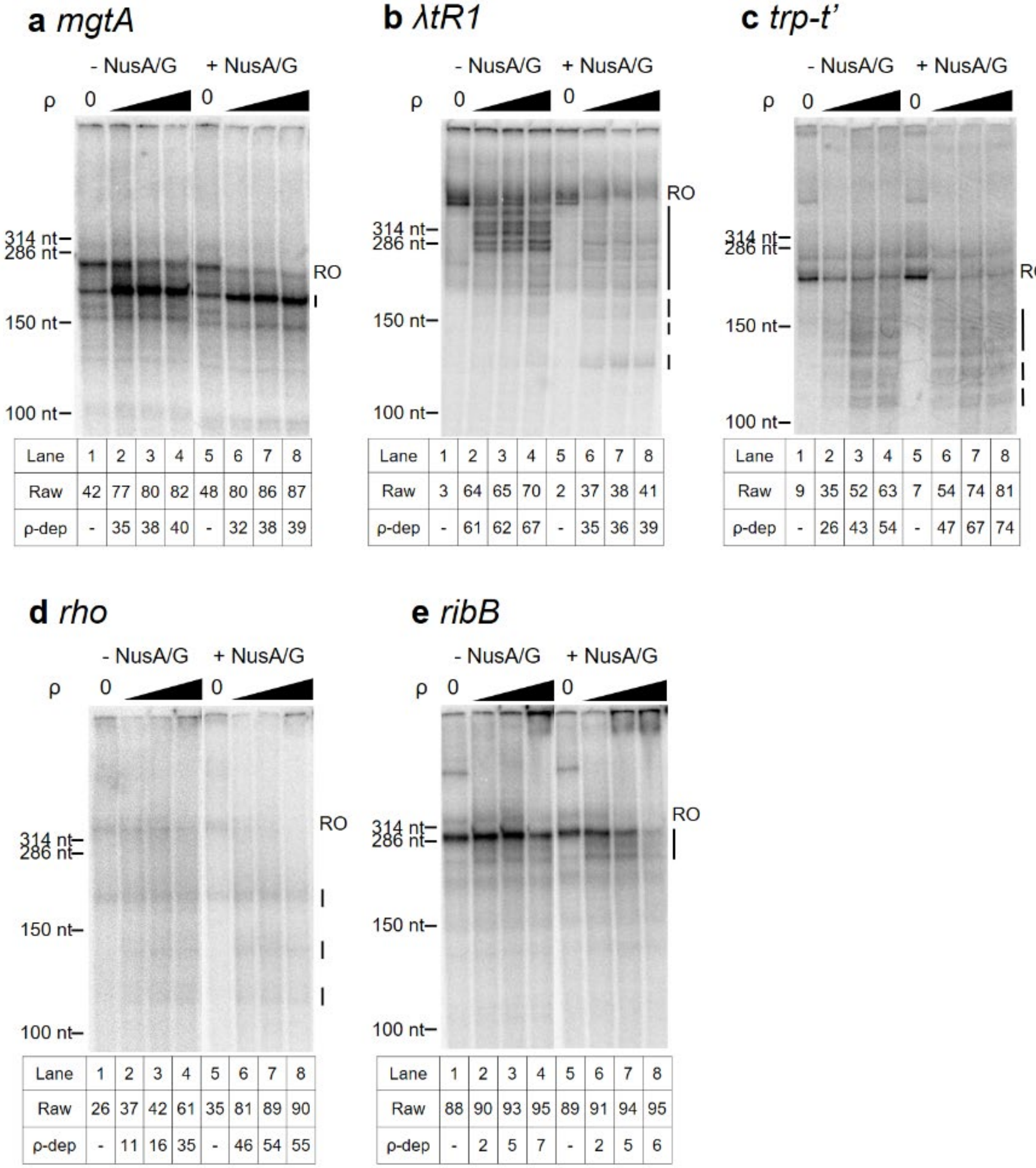

a *mgtA*

Lane	1	2	3	4	5	6	7	8
Raw	42	77	80	82	48	80	86	87
ρ-dep	-	35	38	40	-	32	38	39

b *λtR1*

Lane	1	2	3	4	5	6	7	8
Raw	3	64	65	70	2	37	38	41
ρ-dep	-	61	62	67	-	35	36	39

c *trp-t'*

Lane	1	2	3	4	5	6	7	8
Raw	9	35	52	63	7	54	74	81
ρ-dep	-	26	43	54	-	47	67	74

d *rho*

Lane	1	2	3	4	5	6	7	8
Raw	26	37	42	61	35	81	89	90
ρ-dep	-	11	16	35	-	46	54	55

e *ribB*

Lane	1	2	3	4	5	6	7	8
Raw	88	90	93	95	89	91	94	95
ρ-dep	-	2	5	7	-	2	5	6

Supplementary Figure 9 | Background termination of five ρ-dependent terminators. Background TEs of five ρ-dependent terminators were measured without ρ. An error bar represents the standard deviation of mean from $n \geq 3$ independent experiments each with >50 molecules. Source data are provided as a Source Data file.

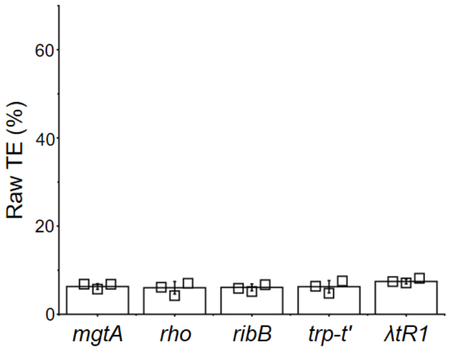

Supplementary Figure 10 | Purification of ρ mutants. Four mutants of *E. coli* ρ were highly purified in soluble forms with a C-terminal His6 tag. Final products (2 μg each) were compared with bovine serum albumin (BSA) and molecular weight standards (15 to 160 kDa) in 12% sodium dodecyl sulphate–polyacrylamide gel electrophoresis. The yields were high, 36% for G51V, 18% for Y80C, 19% for P279L, and 33% for P279S. The G51V, Y80C, and P279S mutants are completely inactive for termination (Fig. 1d), while the P279L mutant is partially active.

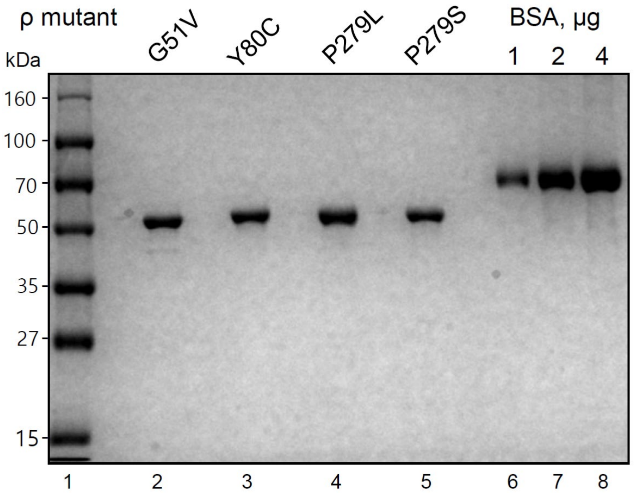

Supplementary Tables

Supplementary Table 1 | Oligonucleotide sequences

1. *mgtA* terminator (314 bp) template

The template was constructed by ligation of A and B1 fragments with AB splint. The mutants for shearing tests were constructed by ligation of A and a B2 with AB. The mutants for displacing tests were constructed by ligation of A and a B3 with AB. Mutations are underlined.

Name	Length	Sequence in the 5' to 3' direction
A	200 nt	TATCAAAAAGAGTATTGACTTAAAGTCTAACCTATAGGATACTTACAGCCATACCGCCA<br>CAAAACTTATGGATTTATGCGTATAATCCGCGGCGCAAATTATTTACTTACCGGAGGCGA<br>CATGGACCCTGAACCCACCCCTCTCCCGCGATGGAGAATTTTCCTTTTCCGGTAAGCCT<br>GCCTCTGCTGTCTTACCGGTGT
B1	114 nt	pGTAAGACAGTGACACAATAACGTCCCTGTTTTTATTTAAACATTGCTCATCGGGCAAG<br>GCTTTGCCGTGCCTGAAGAATTTTCTGCGCCTGACTTCGGCGCGGAGGGATTACCT
AB	40 nt	TTATTGTGTCACTGTCTTACACACCGGTAAGACAGCAGAG
B2(1)<br>(AU11)	114 nt	pGTAAGACAGTGACACAATAACGTCCCTGTTTTTATTTAAACATTGCTCATCGGGCAAG<br>GCGGGGCCGTGCCTGAAGAATTTTCTGCGCCTGACTTCGGCGCGGAGGGATTACCT
B2(2)<br>(AU56)	114 nt	pGTAAGACAGTGACACAATAACGTCCCTGTTTTTATTTAAACATTGCTCATCGGGCAAG<br>GTTTTGCCGTGCCTGAAGAATTTTCTGCGCCTGACTTCGGCGCGGAGGGATTACCT
B2(3)<br>(AU67)	114 nt	pGTAAGACAGTGACACAATAACGTCCCTGTTTTTATTTAAACATTGCTCATCGGGCAAG<br>GCTTTTTCGTGCCTGAAGAATTTTCTGCGCCTGACTTCGGCGCGGAGGGATTACCT
B2(4)<br>(AU78)	114 nt	pGTAAGACAGTGACACAATAACGTCCCTGTTTTTATTTAAACATTGCTCATCGGGCAAG<br>GTTTTTTCGTGCCTGAAGAATTTTCTGCGCCTGACTTCGGCGCGGAGGGATTACCT
B2(5)<br>(AU100)	114 nt	pGTAAGACAGTGACACAATAACGTCCCTGTTTTTATTTAAACATTGCTCATCGGGCAAG<br>GTTTTTTATTGCCTGAAGAATTTTCTGCGCCTGACTTCGGCGCGGAGGGATTACCT
B3(1)<br>(nt16)	114 nt	pGTAAGACAGTGACACAATAACGTCCCTGTTTTTATTTAAACATTGCTCATCGGGCAAG<br>GCTTTGCCGTGCCTGAAGAATTTTCACGGCCTGACTTCGGCGCGGAGGGATTACCT
B3(2)<br>(nt1)	114 nt	pGTAAGACAGTGACACAATAACGTCCCTGTTTTTATTTAAACATTGCTCATCGGGCAAG<br>GCTTTGCCGACGCTGAAGAATTTTCTGCGCCTGACTTCGGCGCGGAGGGATTACCT
B3(3)<br>(nt3)	114 nt	pGTAAGACAGTGACACAATAACGTCCCTGTTTTTATTTAAACATTGCTCATCGGGCAAG<br>GCTTTGCGCAGCCTGAAGAATTTTCTGCGCCTGACTTCGGCGCGGAGGGATTACCT
B3(4)<br>(nt5)	114 nt	pGTAAGACAGTGACACAATAACGTCCCTGTTTTTATTTAAACATTGCTCATCGGGCAAG<br>GCTTTCGGGTGCCTGAAGAATTTTCTGCGCCTGACTTCGGCGCGGAGGGATTACCT
B3(5)<br>(nt7)	114 nt	pGTAAGACAGTGACACAATAACGTCCCTGTTTTTATTTAAACATTGCTCATCGGGCAAG<br>GCTAACCCGTGCCTGAAGAATTTTCTGCGCCTGACTTCGGCGCGGAGGGATTACCT
B3(6)<br>(nt9)	114 nt	pGTAAGACAGTGACACAATAACGTCCCTGTTTTTATTTAAACATTGCTCATCGGGCAAG<br>GGAATGCCGTGCCTGAAGAATTTTCTGCGCCTGACTTCGGCGCGGAGGGATTACCT
B3(7)<br>(nt11)	114 nt	pGTAAGACAGTGACACAATAACGTCCCTGTTTTTATTTAAACATTGCTCATCGGGCAAC<br>CGTTTGCCGTGCCTGAAGAATTTTCTGCGCCTGACTTCGGCGCGGAGGGATTACCT
B3(8)<br>(nt13)	114 nt	pGTAAGACAGTGACACAATAACGTCCCTGTTTTTATTTAAACATTGCTCATCGGGCTTC<br>GCTTTGCCGTGCCTGAAGAATTTTCTGCGCCTGACTTCGGCGCGGAGGGATTACCT

B3(9)<br>(nt15)	114 nt	5pGTAAGACAGTGACACAATAACGTCCCTGTTTTTATTTAAACATTGCTCATCGGCGTAG<br>GCTTTGCCGTGCCTGAAGAATTTTCTGCGCCTGACTTCGGCGCGGAGGGATTACCT
B3(10)<br>(nt17)	114 nt	pGTAAGACAGTGACACAATAACGTCCCTGTTTTTATTTAAACATTGCTCATCCCCCAAG<br>GCTTTGCCGTGCCTGAAGAATTTTCTGCGCCTGACTTCGGCGCGGAGGGATTACCT
B3(11)<br>(nt19)	114 nt	pGTAAGACAGTGACACAATAACGTCCCTGTTTTTATTTAAACATTGCTCAAGCGGCAAG<br>GCTTTGCCGTGCCTGAAGAATTTTCTGCGCCTGACTTCGGCGCGGAGGGATTACCT
B3(12)<br>(nt21)	114 nt	pGTAAGACAGTGACACAATAACGTCCCTGTTTTTATTTAAACATTGCTGTACGGGCAAG<br>GCTTTGCCGTGCCTGAAGAATTTTCTGCGCCTGACTTCGGCGCGGAGGGATTACCT
B3(13)<br>(nt23)	114 nt	pGTAAGACAGTGACACAATAACGTCCCTGTTTTTATTTAAACATTGGAGATCGGGCAAG<br>GCTTTGCCGTGCCTGAAGAATTTTCTGCGCCTGACTTCGGCGCGGAGGGATTACCT
B3(14)<br>(nt25)	114 nt	pGTAAGACAGTGACACAATAACGTCCCTGTTTTTATTTAAACATACGTCATCGGGCAAG<br>GCTTTGCCGTGCCTGAAGAATTTTCTGCGCCTGACTTCGGCGCGGAGGGATTACCT
B3(15)<br>(nt41)	114 nt	pGTAAGACAGTGACACAATAACGTCCCTCAATTTATTTAAACATTGCTCATCGGGCAAG<br>GCTTTGCCGTGCCTGAAGAATTTTCTGCGCCTGACTTCGGCGCGGAGGGATTACCT

2. *rho* terminator (390 bp) template

The template was constructed by ligation of rho-A and rho-B fragments with rho-AB splint.

Name	Length	Sequence in the 5' to 3' direction
rho-A	200 nt	TATCAAAAAGAGTATTGACTTAAAGTCTAACCTATAGGATACTTACAGCCATACCCCTA<br>AATTGTCAGGATCTCTGGACGCCCGGTCTGAGTCGTGCTAAGTTAGTATTGACTTCGAA<br>TTAAACATACCTTATTAAGTTTGAATCTGGTTTTATCCGTCACTTCCCGTTTTTTCTCGCA<br>CGAGAAGTGGAAAGATTCCTG
rho-B	190 nt	pGCTCTTCGCTCATTCCGTCTTGTCGTTTCAGTTCTGCGTACTTTCCTGTGACCAGACA<br>GCGAACAGACATGAGTTGATAGCCGTAAACAGGCATGGATGACCCTGCCATACCATTC<br>ACAACATTAAGTTCGAGATTTACCCCAAGTTTAAGAACTCACACCATTATGAATCTTAC<br>CGAATTAAAGAATAC
rho-AB	40 nt	AGACGGAATGAGCGAAGAGCCAGGAATCTTTCCACTTCTC

3. *ribB* terminator (350 bp) template

The template was constructed by ligation of rib-A and rib-B fragments with rib-AB splint.

Name	Length	Sequence in the 5' to 3' direction
rib-A	200 nt	TATCAAAAAGAGTATTGACTTAAAGTCTAACCTATAGGATACTTACAGCCATCCACCCT<br>CAGGGCGGGGCGAAATTCCCCACCGGCGGTAAATCAACTCAGTTGAAAGCCCGCGAG<br>CGCTTTGGGTGCGAACTCAAAGGACAGCAGATCCGGTGTAATTCCGGGGCCGACGGT<br>TAGAGTCCGGATGGGAGAGAGTAACGA3’
rib-B	150 nt	pTTCTGTCGGGCATGGACCCGCTCACGTTATTTTGGCTATATGCCGCCACTCCTAAGACT<br>GCCCTGATTCTGGTAACCATAATTTAGTGAGGTTTTTTACCATGAATCAGACGCTACT<br>TTCCTCTTTTGGTACGCCTTTCGAACGTGTT
rib-AB	40 nt	CGGGTCCATGCCCGACAGAATCGTTACTCTCTCCCATCCG

4. *trp t'* terminator (286 bp) template

The template was constructed by ligation of trp-A and trp-B fragments with trp-AB splint.

Name	Length	Sequence in the 5' to 3' direction
trp-A	200 nt	TATCAAAAAGAGTATTGACTTAAAGTCTAACCTATAGGATACTTACAGCCATACGCCGG TCGAACGTCAACTTACGTCATTTTTCCGCCCAACAGTAATATAATCAAACAAATTAATC CCGCAACATAACACCAGTAAAATCAATAATTTCTCTAAGTCACTTATTCCTCAGGTAAT TGTTAATATATCCAGAATGTTC
trp-B	86 nt	pCTCAAAATATATTTTCCCTCTATCTTCTCGTTGCGCTTAATTTGACTAATTCTCATTAGC GACTAATTTTAATGAGTGTCGACACA
trp-AB	40 nt	GAGGGAAAATATATTTTGAGGAACATTCTGGATATATTAA

5. λ *tR1* terminator (548 bp) template

The template was constructed by ligation of tr1-A, tr1-B and tr1-C fragments with tr1-AB and tr1-BC splints.

Name	Length	Sequence in the 5' to 3' direction
tr1-A	200 nt	TATCAAAAAGAGTATTGACTTAAAGTCTAACCTATAGGATACTTACAGCCATCAAGTAA GGAGGTTGTATGGAACAACGCATAACCCTGAAAGATTATGCAATGCGCTTTGGGCAAA CCAAGACAGCTAAAGATCTCGGCGTATATCAAAGCGCGATCAACAAGGCCATTCATGC AGGCCGAAAGATTTTTTTAACTATA
tr1-B	200 nt	pAACGCTGATGGAAGCGTTTATGCGGAAGAGGTAAAGCCCTTCCCGAGTAACAAAAA AACAACAGCATAAATAACCCCGCTCTTACACATTCCAGCCCTGAAAAAGGGCATCAAA TTAAACCACACCTATGGTGTATGCATTTATTTGCATACATTCAATCAATTGTTATCTAAGG AAATACTTACATATGGTTCGTGCAA
tr1-C	148 nt	pACAAACGCAACGAGGCTCTACGAATCGAGAGTGCGTTGCTTAACAAAATCGCAATGC TTGGAACTGAGAAGACAGCGGAAGCTGTGGGCGTTGATAAGTCGCAGATCAGCAGGT GGAAGAGGGACTGGATTCCAAAGTTCTCAATGCT
tr1-AB	40 nt	TAAACGCTTCCATCAGCGTTTATAGTTAAAAAAATCTTTC
tr1-BC	40 nt	TAGAGCCTCGTTGCGTTTGTTTGCACGAACCATATGTAAG

The ligation products were amplified by PCR using a common biotin-labeled forward primer 5'TATCAAAAAGAGTATTG ACTTAAAGTCTAA and a respective Cy5-labeled backward primer, 5'AGGTAATCCCTCCGC for *mgtA* terminator, 5'AGCATTGAGAACTTTGGAATC for λ *tR1* terminator, 5'TGTGTCGACACTCATTAAAATTAG for *trp t'* terminator, 5'GTATTCTTTAATTCGGTAAGATTCATAATG for *rho* terminator, or 5'AACACGTTCGAAAGGC for *ribB* terminator.

Supplementary Table 2 | Termination efficiencies (TEs)

ρ-dependent TEs were estimated by subtracting the background TE (6.3%) from raw TEs.

Normalized TEs were estimated by dividing ρ-dependent TEs with (100% - background TE).

n means the number of molecules analyzed.

1. ρ-dependent TEs of *mgtA* terminator at varying concentrations of ρ (holistic assay, Fig. 1d)

[ρ] (nM)	Assay	Decomposing termination (%)	Recycling termination (%)	*n* in replicated experiments
0.5	Holistic	9.3 ± 0.8	0.0 ± 0.0	301 = 68 + 116 + 117
1	Holistic	9.9 ± 2.1	0.0 ± 0.0	303 = 75 + 105 + 123
5	Holistic	32.5 ± 2.1	4.8 ± 2.1	250 = 58 + 75 + 117
10	Holistic	43.6 ± 5.3	6.2 ± 4.2	403 = 190 + 100 + 113
50	Holistic	42.4 ± 4.5	8.6 ± 5.3	304 = 132 + 105 + 67
100	Holistic	42.5 ± 4.0	8.6 ± 2.0	592 = 226 + 108 + 65 + 70 + 123
200	Holistic	47.1 ± 2.5	7.0 ± 4.3	358 = 66 + 92 + 125 + 75

2. ρ-dependent TEs of *mgtA* terminator at varying concentrations of ρ (stand-by assay, Fig. 1f)

Condition	Decomposing termination (%)	Recycling termination (%)	*n* in replicated experiments
ρ [a]	42.5 ± 4.0	8.6 ± 2.0	592 = 226 + 108 + 65 + 70 + 123
-ρ [b]	6.3 ± 0.7	0.0 ± 0.0	413 = 103 + 117 + 193
ρ + inhibitor [c]	7.6 ± 1.0	0.0 ± 0.0	210 = 37 + 90 + 83
ρ^{P279S} [d]	7.5 ± 1.7	0.0 ± 0.0	279 = 120 + 83 + 76
ρ^{G51V} [d]	7.6 ± 1.0	0.0 ± 0.0	290 = 108 +63 + 119
ρ^{Y80C} [d]	6.9 ± 0.8	0.0 ± 0.0	219 = 56 + 80 + 83

[a] Holistic assay ([ρ] = 100 nM)

[b] Transcription without ρ

[c] Holistic assay ([ρ] = 100 nM, and [bicyclomycin] = 1 mM)

[d] Holistic assay with a ρ mutant at [ρ] = 100 nM

3. ρ-dependent TEs of *mgtA* terminator at varying concentrations of ρ (stand-by assay, Fig. 2b)

[ρ] (nM)	Assay	Decomposing termination (%)	Recycling termination (%)	*n* in replicated experiments
0.5	Stand-by	6.3 ± 1.0	0.0 ± 0.0	247 = 93 + 77 + 77
1	Stand-by	8.0 ± 0.8	0.0 ± 0.0	224 = 65 + 68 + 91
5	Stand-by	8.1 ± 2.0	0.0 ± 0.0	417 = 85 + 54 +199 + 79
10	Stand-by	8.7 ± 1.0	0.0 ± 0.0	246 = 53 +91 +102
50	Stand-by	11.0 ± 0.7	0.3 ± 0.5	336 = 123 + 99 + 114
100	Stand-by	15.8 ± 0.8	0.2 ± 0.3	420 = 91 +73 + 79 +177
200	Stand-by	17.0 ± 1.4	0.0 ± 0.0	326 = 168 + 72 + 86

4. ρ-dependent TEs of *mgtA* terminator at varying concentrations of ρ (catch-up assay, Fig. 2e)

[ρ] (nM)	Assay	Decomposing termination (%)	Recycling termination (%)	*n* in replicated experiments
0.5	Catch-up	7.6 ± 1.8	1.3 ± 2.3	317 = 125 + 91 + 101
1	Catch-up	9.5 ± 1.2	0.0 ± 0.0	241 = 68 + 95 + 78
5	Catch-up	8.3 ± 1.1	1.7 ± 1.3	302 = 90 + 117 + 95
10	Catch-up	14.0 ± 1.2	0.5 ± 1.0	207 = 57 + 75 + 75
50	Catch-up	23.8 ± 2.7	3.1 ± 3.0	259 = 56 + 106 + 97
100	Catch-up	23.4 ± 3.7	5.9 ± 3.8	236 = 83 + 82 +71
200	Catch-up	24.5 ± 4.8	4.3 ± 3.8	588 = 133 + 86 + 109 + 110 + 150

5. ρ-dependent TEs of various terminators (Fig. 3a, Fig. 3b)

Terminator	Assay	Decomposing termination (%)	Recycling termination (%)	*n* in replicated experiments
mgtA	Holistic	42.5 ± 4.0	8.6 ± 2.0	592 = 226 + 108 + 65 + 70 + 123
	Stand-by	15.8 ± 0.8	0.2 ± 0.3	420 = 91 +73 + 79 +177
	Catch-up	23.4 ± 3.7	5.9 ± 3.8	236 = 83 + 82 +71
rho	Holistic	22.1 ± 3.1	6.1 ± 1.9	278 = 136 + 65 +77
	Stand-by	10.7 ± 2.3	0.0 ± 0.0	221 = 55 + 95 + 67
	Catch-up	27.8 ± 4.7	5.9 ± 3.8	465 = 256 + 146 + 63
ribB	Holistic	9.3 ± 2.8	3.0 ± 2.7	364 = 129 + 143 + 92
	Stand-by	6.2 ± 1.8	0.0 ± 0.0	301 = 136 + 88 +77
	Catch-up	10.0 ± 2.8	1.3 ± 1.2	305 = 93 +79 + 133
trp-t'	Holistic	30.2 ± 1.8	11.3 ± 3.6	406 = 200 + 69 + 137
	Stand-by	3.8 ± 2.7	0.5 ± 0.9	420 = 248 + 116 + 56
	Catch-up	26.5 ± 2.0	9.0 ± 2.7	412 = 130 + 150 + 132
λtR1	Holistic	32.5 ± 2.9	4.1 ± 1.4	243 = 77 + 108 + 58
	Stand-by	0.4 ± 0.6	0.0 ± 0.0	422 = 167 + 86 + 65 + 104
	Catch-up	30.5 ± 1.2	4.1 ± 0.5	416 = 161 + 62 + 114 + 79
mgtA (+NusA/G)	Holistic	28.0 ± 3.0	6.8 ± 3.3	399 = 113 + 133 + 153
	Stand-by	10.1 ± 2.5	0.0 ± 0.0	553 = 107 + 151 + 123 + 172
	Catch-up	11.5 ± 2.0	1.7 ± 1.8	473 = 143 + 129 + 88 + 113

6. Normalized ρ-dependent TEs of various hybrid-shearing mutants of *mgtA* terminator (stand-by assay, Fig. 4c, Fig. 4d)

Substrate	Assay	Decomposing termination (%)	Recycling termination (%)	*n* in replicated experiments
AU11	Stand-by	7.3 ± 1.7	0.2 ± 0.3	592 = 229 + 123 + 240
AU44 (WT)	Stand-by	16.9 ± 0.8	0.2 ± 0.3	420 = 91 + 73 + 79 + 177
AU55	Stand-by	6.8 ± 1.7	0.0 ± 0.0	491 = 152 + 153 + 186
AU66	Stand-by	11.2 ± 1.1	0.0 ± 0.0	413 = 152 + 98 + 163
AU77	Stand-by	14.4 ± 0.9	0.0 ± 0.0	536 = 246 + 137 + 153
AU100	Stand-by	0.7 ± 1.3	0.0 ± 0.0	571 = 251 + 180 + 140

7. Normalized ρ-dependent TEs of various hybrid-shearing mutants of *mgtA* terminator (catch-up assay, Fig. 4c, Fig. 4d)

Substrate	Assay	Decomposing termination (%)	Recycling termination (%)	*n* in replicated experiments
AU11	Catch-up	26.3 ± 1.6	5.5 ± 0.6	488 = 166 + 172 + 150
AU44 (WT)	Catch-up	24.9 ± 4.0	5.9 ± 3.8	236 = 83 +82 + 71
AU55	Catch-up	19.5 ± 1.1	7.9 ± 1.9	493 = 166 + 191 + 136
AU66	Catch-up	11.4 ± 0.9	9.1 ± 0.8	570 = 153 + 144 + 108 + 165
AU77	Catch-up	29.3 ± 1.2	12.2 ± 1.5	576 = 166 + 242 + 168
AU100	Catch-up	20.2 ± 2.7	11.9 ± 0.7	385 = 148 + 143 +94

8. Normalized ρ-dependent TEs of various nontemplate strand mutants of *mgtA* terminator (stand-by assay, Fig. 5b, Fig. 5c)

Substrate	Assay	Decomposing termination (%)	Recycling termination (%)	*n* in replicated experiments
nt40 (-40)	Stand-by	17.0 ± 1.1	0.0 ± 0.0	395 = 181 + 95 + 119
nt24 (-24)	Stand-by	16.3 ± 2.0	0.0 ± 0.0	363 = 140 + 128 + 95
nt22 (-22)	Stand-by	13.2 ± 1.0	0.0 ± 0.0	297 = 97 + 144 + 56
nt20 (-20)	Stand-by	17.9 ± 1.1	0.0 ± 0.0	225 = 67 + 62 + 96
nt18 (-18)	Stand-by	15.5 ± 1.3	0.0 ± 0.0	250 = 71 + 97 + 82
nt16 (-16)	Stand-by	17.1 ± 1.5	0.0 ± 0.0	360 = 128 + 155 + 77
nt14 (-14)	Stand-by	14.6 ± 1.4	0.0 ± 0.0	298 = 96 + 99 + 103
nt12 (-12)	Stand-by	1.8 ± 1.5	0.0 ± 0.0	301 = 127 + 98 + 76
nt10 (-10)	Stand-by	8.9 ± 1.6	0.0 ± 0.0	332 = 172 + 101 + 59
nt8 (-8)	Stand-by	9.0 ± 2.2	0.0 ± 0.0	327 = 146 + 128 + 53
nt6 (-6)	Stand-by	8.4 ± 1.1	0.0 ± 0.0	483 = 158 + 167 + 158
nt4 (-4)	Stand-by	17.0 ± 1.2	0.0 ± 0.0	363 = 100 + 119 + 144
nt2 (-2)	Stand-by	16.7 ± 2.0	0.0 ± 0.0	284 = 104 + 60 + 51 + 69
nt1 (+1)	Stand-by	16.8 ± 1.5	0.0 ± 0.0	338 = 115 + 120 + 103
nt17 (+17)	Stand-by	13.8 ± 1.6	0.0 ± 0.0	271 = 65 + 110 + 96

9. Normalized ρ-dependent TEs of various nontemplate strand mutants of *mgtA* terminator (catch-up assay, Fig. 5b, Fig. 5c)

Substrate	Assay	Decomposing termination (%)	Recycling termination (%)	*n* in replicated experiments
nt40 (-40)	Catch-up	24.6 ± 0.7	4.3 ± 0.9	372 = 93 + 156 + 123
nt24 (-24)	Catch-up	20.2 ± 2.2	3.7 ± 2.5	428 = 173 + 93 + 162
nt22 (-22)	Catch-up	18.6 ± 1.5	4.5 ± 2.8	247 = 67 + 78 + 102
nt20 (-20)	Catch-up	26.7 ± 1.2	4.9 ± 0.5	266 = 84 + 112 + 70
nt18 (-18)	Catch-up	25.8 ± 1.5	4.3 ± 0.9	369 = 108 + 168 + 93
nt16 (-16)	Catch-up	27.0 ± 2.6	5.7 ± 0.9	280 = 115 + 89 + 76
nt14 (-14)	Catch-up	28.5 ± 5.3	4.7 ± 0.1	380 = 123 + 148 + 109
nt12 (-12)	Catch-up	9.0 ± 2.3	5.3 ± 0.8	285 = 123 + 97 + 65
nt10 (-10)	Catch-up	19.2 ± 3.4	4.6 ± 0.9	370 = 144 + 121 + 105
nt8 (-8)	Catch-up	22.8 ± 2.2	4.9 ± 0.5	445 = 121 + 145 + 109 + 70

nt6 (-6)	Catch-up	23.4 ± 0.5	4.8 ± 0.9	314 = 175 + 88 + 51
nt4 (-4)	Catch-up	26.2 ± 1.5	6.2 ± 0.7	421 = 147 +149 + 125
nt2 (-2)	Catch-up	23.5 ± 1.9	5.7 ± 3.2	299 = 97 + 109 + 93
nt1 (+1)	Catch-up	24.0 ± 1.1	5.4 ± 0.2	221 = 76 +70 + 75
nt17 (+17)	Catch-up	22.0 ± 1.4	6.1 ± 3.6	380 = 117 + 123 + 140

Supplementary Table 3 | Readthrough and termination timings (Fig. 3d-e)

n means the number of molecules analyzed.

mgtA+Nus indicates *mgtA* terminator in the presence of NusA and NusG.

(1-1) Decomposing termination timing in holistic assay

Terminator	Assay	Pathway	Timing (s)	*n* in replicated experiments
mgtA	Holistic	Decomposing	199 ± 32.4	289 = 113 + 49 + 36 + 34 + 57
mgtA+Nus	Holistic	Decomposing	241 ± 26.1	146 = 45 + 83 + 53
rho	Holistic	Decomposing	147 ± 10.2	78 = 35 + 19 + 24
ribB	Holistic	Decomposing	596 ± 16.1	56 = 21 + 24 + 11
trp-t'	Holistic	Decomposing	153 ± 19.3	148 = 71 + 26 + 51
λtR1	Holistic	Decomposing	240 ± 28.2	97 = 32 + 44 + 21

(1-2) Recycling termination timing in holistic assay

Terminator	Assay	Pathway	Timing (s)	*n* in replicated experiments
mgtA	Holistic	Recycling	27 ± 1.1	51 = 16 + 12 + 5 + 8 + 10
mgtA+Nus	Holistic	Recycling	33 ± 1.7	27 = 10 + 4 + 13
rho	Holistic	Recycling	20 ± 1.2	17 = 6 + 5 + 6
ribB	Holistic	Recycling	42 ± 2.9	11 = 3 + 2 + 6
trp-t'	Holistic	Recycling	7 ± 0.2	46 = 26 + 9 + 11
λtR1	Holistic	Recycling	16 ± 1.6	10 = 2 + 5 + 3

(1-3) Readthrough timing in holistic assay

Terminator	Assay	Pathway	Timing (s)	*n* in replicated experiments
mgtA	Holistic	Readthrough	198 ± 9.2	252 = 97 + 47 + 24 + 28 + 56
mgtA+Nus	Holistic	Readthrough	164 ± 13.9	226 = 58 + 81 + 87
rho	Holistic	Readthrough	92 ± 9.6	183 = 95 + 41 + 47
ribB	Holistic	Readthrough	73 ± 7.9	297 = 105 + 117 + 75
trp-t'	Holistic	Readthrough	77 ± 5.4	212 = 100 + 37 + 52
λtR1	Holistic	Readthrough	146 ± 18.8	136 = 43 + 59 + 34

(2-1) Decomposing termination timing in stand-by assay

Terminator	Assay	Pathway	Timing (s)	*n* in replicated experiments
mgtA	Stand-by	Decomposing	322 ± 71.3	93 = 20 + 16 + 18 + 39
mgtA+Nus	Stand-by	Decomposing	421 ± 35.2	103 = 18 + 25 + 25 + 32
rho	Stand-by	Decomposing	157 ± 17.4	37 = 8 + 17 + 12
ribB	Stand-by	Decomposing	504 ± 95.5	37 = 16 + 10 + 11
trp-t'	Stand-by	Decomposing	245 ± 33.3	42 = 22 + 15 + 5
λtR1	Stand-by	Decomposing	160 ± 44.8	33 = 13 + 7 + 5 + 8

(2-2) Recycling termination timing in stand-by assay

Terminator	Assay	Pathway	Timing (s)	*n* in replicated experiments
mgtA	Stand-by	Recycling	Not measurable	0 = 0 + 0 + 0 + 1
mgtA+Nus	Stand-by	Recycling	Not measurable	0 = 0 + 0 + 0 + 0
rho	Stand-by	Recycling	Not measurable	0 = 0 + 0 + 0
ribB	Stand-by	Recycling	Not measurable	0 = 0 + 0 + 0
trp-t'	Stand-by	Recycling	Not measurable	2 = 1 + 0 + 1
λtR1	Stand-by	Recycling	Not measurable	0 = 0 + 0 + 0 + 0

(2-3) Readthrough timing in stand-by assay

Terminator	Assay	Pathway	Timing (s)	*n* in replicated experiments
mgtA	Stand-by	Readthrough	137 ± 3.3	326 = 71 + 57 + 61 + 137
mgtA+Nus	Stand-by	Readthrough	132 ± 14.3	450 = 89 + 126 + 98 + 137
rho	Stand-by	Readthrough	91 ± 10.0	184 = 47 + 82 + 55
ribB	Stand-by	Readthrough	113 ± 11.2	264 = 120 + 78 + 66
trp-t'	Stand-by	Readthrough	78 ± 7.0	376 = 225 + 101 + 50
λtR1	Stand-by	Readthrough	61 ± 4.9	389 = 154 + 79 + 60 +96

(3-1) Decomposing termination timing in catch-up assay

Terminator	Assay	Pathway	Timing (s)	*n* in replicated experiments
mgtA	Catch-up	Decomposing	189 ± 20.9	70 = 22 + 24 + 24
mgtA+Nus	Catch-up	Decomposing	379 ± 37.2	95 = 26 + 27 + 18 + 24
rho	Catch-up	Decomposing	246 ± 43.1	157 = 91 + 49 + 17
ribB	Catch-up	Decomposing	260 ± 26.2	49 = 16 + 10 + 23
trp-t'	Catch-up	Decomposing	209 ± 29.0	135 = 44 + 50 + 41
λtR1	Catch-up	Decomposing	132 ± 12.9	158 = 60 + 23 + 44 + 31

(3-2) Recycling termination timing in catch-up assay

Terminator	Assay	Pathway	Timing (s)	*n* in replicated experiments
mgtA	Catch-up	Recycling	21 ± 2.0	14 = 6 + 7 + 1
mgtA+Nus	Catch-up	Recycling	26 ± 2.1	8 = 1 + 2 + 4 + 1
rho	Catch-up	Recycling	27 ± 1.5	6 = 2 + 2 + 2
ribB	Catch-up	Recycling	45 ± 5.8	6 = 1 + 3 + 2
trp-t'	Catch-up	Recycling	13 ± 0.4	37 = 9 + 12 + 16
λtR1	Catch-up	Recycling	23 ± 0.6	17 = 6 + 3 + 5 + 3

(3-3) Readthrough timing in catch-up assay

Terminator	Assay	Pathway	Timing (s)	*n* in replicated experiments
mgtA	Catch-up	Readthrough	177 ± 8.8	152 = 55 + 51 + 46
mgtA+Nus	Catch-up	Readthrough	167 ± 11.9	370 = 116 + 100 + 66 + 88
rho	Catch-up	Readthrough	231 ± 9.5	302 = 163 + 95 + 44
ribB	Catch-up	Readthrough	90 ± 10.0	250 = 76 + 66 + 108
trp-t'	Catch-up	Readthrough	141 ± 21.9	240 = 77 + 88 + 75
λtR1	Catch-up	Readthrough	112 ± 15.9	241 = 95 + 36 + 65 + 45

Supplementary Table 4 | Statistics for readthrough and termination timings (Fig. 3d)

n means the sample size.

σ means standard deviation.

Assay	Pathway	E[X]	σ	*n*	σ^2/sqrt(n)
Catch-up	Readthrough	153.1	50.24	6	40.87
Catch-up	Decomposing	235.7	83.46	6	1160.97
Catch-up	Recycling	25.8	10.71	6	19.11
Stand-by	Readthrough	102.0	30.71	6	153.01
Stand-by	Decomposing	301.7	30.30	6	3322.65
